# A novel statistical method for assessing effective adherence to medication and calculating optimal drug dosages

**DOI:** 10.1371/journal.pone.0195663

**Published:** 2018-04-20

**Authors:** Garrett Greene, Richard W. Costello, Breda Cushen, Imran Sulaiman, Elaine Mac Hale, Ronan M. Conroy, Frank Doyle

**Affiliations:** 1 Division of Population Health Sciences, Royal College of Surgeons in Ireland, Dublin, Ireland; 2 Clinical Research Centre, Beaumont Hospital, Dublin, Ireland; 3 Department of Medicine, Royal College of Surgeons in Ireland, Dublin, Ireland; Lee Kong Chian School of Medicine, SINGAPORE

## Abstract

**Objective:**

We derive a novel model-based metric for effective adherence to medication, and validate it using data from the INhaler Compliance Assessment device (INCA^TM^). This technique employs dose timing data to estimate the threshold drug concentration needed to maintain optimal health.

**Methods:**

The parameters of the model are optimised against patient outcome data using maximum likelihood methods. The model is fitted and validated by secondary analysis of two independent datasets from two remote-monitoring studies of adherence, conducted through clinical research centres of 5 Irish hospitals. Training data came from a cohort of asthma patients (~ 47,000 samples from 218 patients). Validation data is from a cohort of 204 patients with COPD recorded between 2014 and 2016.

**Results:**

The time above threshold measure is strongly predictive of adverse events (exacerbations) in COPD patients (Odds Ratio of exacerbation = 0.52 per SD increase in adherence, 95% Confidence Interval [0.34–0.79]). This compares well with the best known previous method, the Area Under the dose-time Curve (AUC) (Odds Ratio = 0.69, 95% Confidence Interval [0.48–0.99]). In addition, the fitted value of the dose threshold (0.56 of prescribed dosage) suggests that prescribed doses may be unnecessarily high given good adherence.

**Conclusions:**

The resulting metric accounts for missed doses, dose-timing errors, and errors in inhaler technique, and provides enhanced predictive validity in comparison to previously used measures. In addition, the method allows us to estimate the correct dosage required to achieve the effect of the medication using the patients’ own adherence data and outcomes. The adherence score does depend not on sex or other demographic factors suggesting that effective adherence is driven by individual behavioural factors.

## Introduction

### The necessity of adherence assessment

Non-Adherence to prescribed treatment regimens is not only a persistent challenge in chronic disease management [1–3], but can also be a confounding factor when interpreting clinical trial results [4]. Good adherence (i.e. correctly following agreed recommendations from a healthcare provider [5, 6]), can include taking medications as directed, increasing physical activity, modifying diet, etc., and is often difficult to ensure or assess.

However the benefits of good adherence are clear: On average, those who adhere to therapy have significantly better health outcomes while non-adherence confers considerable costs on healthcare providers and patients [1, 3, 5, 7–10]. Consider the example of Chronic Obstructive Pulmonary Disease (COPD).While inhaled bronchodilator drugs are efficacious in reducing the rate of exacerbations of COPD and improving exercise capacity and quality of life [11, 12], estimates of adherence in COPD vary widely depending on the definitions and measures used. Reported rates are 70–90% in clinical drug trials to 20–60% in observational studies [8, 13, 14]. Despite the overall evidence for long-acting inhaled therapy from clinical trials, there remains a significant gap between efficacy and effectiveness [15]. A recent systematic review has demonstrated that, although studies were heterogeneous, non-adherence in COPD was associated with mortality, increased hospitalisations, impaired quality of life and reduced productivity [8]. Thus, promoting adherence to appropriate medication is an important clinical goal.

### Measuring adherence in a clinical setting

Assessing adherence to medication is an essential element both in the ongoing management of chronic disease and in the development of novel treatments. Indeed, in a clinical setting, any attempt to determine the effectiveness of a medication on an individual patient can be hopelessly confounded by a lack of accurate adherence information, potentially leading to over-prescription or unnecessary additional treatment [16, 17]. This is particularly true in the case of inhaled medications, where errors in inhaler technique can result in the patient receiving a drastically reduced dose, unbeknownst to clinicians [18, 19].

In recent years, adherence has been codified using the ABC Taxonomy [20], which defines three stages of adherence; initiation, implementation and discontinuation. In this paper we consider patients receiving ongoing treatment for chronic illness, and as a result we will primarily address the implementation element, namely, the regularity and proficiency with which patients take their inhaler.

There are several established ways to assess implementation adherence to inhaled medications [1, 3, 5, 6, 21–23]. However, each of these methods has significant drawbacks. Perhaps the most widely used is patient self-report which has been shown to be susceptible to reporting biases, with little or no correlation between self-report and objective adherence measures [23–26]. Alternatively, medication checks such as pharmacy reconciliation, refill rates and drug counter checks can be performed to assess adherence [3, 4, 14, 23]. Although these methods have their own strengths, they do not provide information about individual behaviours, such as when and how medicine is actually taken, and cannot detect certain behaviours such as medication dumping or sharing, which negatively affect actual adherence. In addition, these measures cannot account for variations in dose-timing. Consequently, they represent, at best, an informative upper-bound for true adherence rates.

#### The INCA device and AUC metric

The effectiveness of many inhaled medications depends critically on correct dose timing as well as on inhaler technique, which must be accounted for in order to assess the patient’s response to treatment [18, 27–29].

The INCA^TM^ device is an acoustic remote-monitoring device for use with dry powder inhalers (Fluticasone/Salmeterol “Seretide” Diskus^TM^), which can record the precise timing of inhaler use, as well as identifying errors in inhaler technique. Using data from this device, Sulaiman et al. recently developed a novel area under the curve (AUC) metric, which combines data on time-of-use with assessment of inhaler technique to account for critical errors. Using remote monitoring data obtained from the INCA^TM^ device, they applied this metric to a cohort of 244 COPD patients [29], and found that, while the mean number of doses taken was 59.8% of expected, once technique errors were accounted for the mean actual adherence rate was only 22.6%.

#### The need to account for dose timing

While the AUC metric mentioned above represents an improvement over standard methods, this and other metrics remain suboptimal. Both standard adherence assessments and recent remote monitoring studies rely on summary scores, such as monthly dose count, monthly AUC [29], or prescription refill rate,with the result that the effects of dose timing are ignored. Furthermore these methods implicitly assume that the effectiveness of treatment is a linear function of the dose received, which is known to be untrue, particularly in the case of steroids [30, 31].

In general, if the aim of medication is to maintain a target concentration of the prescribed drug in the system [30, 32–34], then we argue that adherence should be defined in terms of the maintained drug concentration, rather than the total number of doses received. Thus, a measure of effective adherence which takes into account the pharmacodynamics of the drug in question may be more effective in predicting outcomes [35]. To demonstrate, in this proof-of-concept paper, we will derive a novel and objectively-derived metric which relies explicitly on dose timing, and can also account for the effects of poor ingestion (in this case, poor inhaler technique). In doing so we aim is to the relationship between precisely-timed patterns of adherence and the associated short-term changes in lung-function and risk of exacerbation, for example, the effect of a delayed dose on subsequent lung function or exacerbation risk over a period of several days. In the following we will:

- Define and mathematically derive a model-based measure of effective adherence
- Fit this model against training data from a cohort of asthma patients
- Validate the model by testing in a COPD sample

## Methods

**Fig 1:**
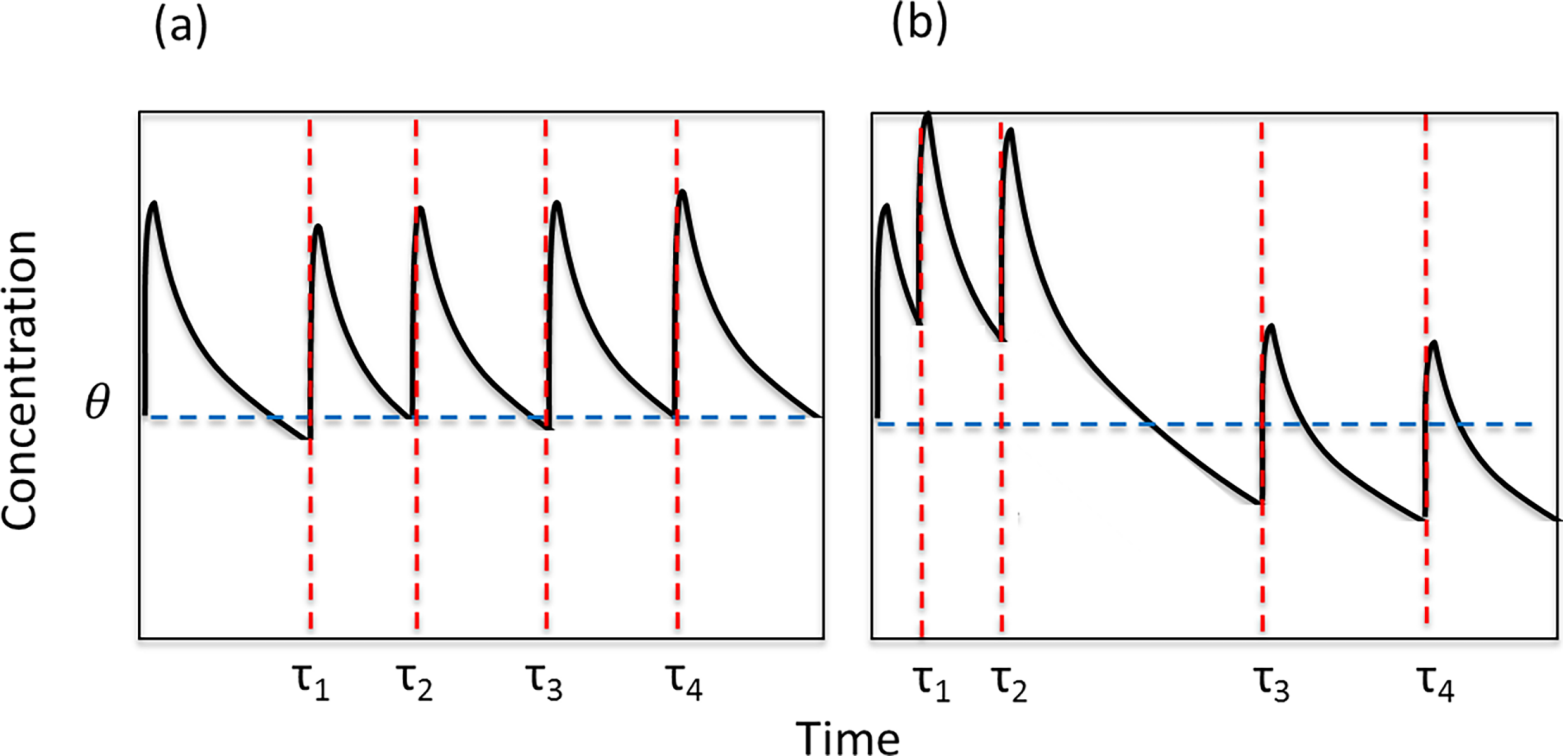
Simulated absorbed ICS concentrations for two patients using Seretide inhalers, with target threshold concentration, *θ*. In both cases the patient has received 4 doses over the period shown. The patient in (a) is more successful in maintaining above-target concentration, while in (b) the concentration drops below target on several occasions.

As mentioned above, if the aim of medication is to maintain a target concentration of the prescribed drug in the system, then the measure of treatment received should be defined in terms of the maintained drug concentration, rather than just the total number of doses received [36]. Fig 1 below shows an illustrative example of how dose timing may affect drug concentration. In both cases the patient has taken the same number of doses over the period shown. However in example (b), the patient displays erratic dose timing, with the result that the concentration drops below target concentration (blue dashed line) for approximately 40% of the period shown.

Therefore, we propose that the adherence rate could be better defined as the proportion of time for which the patient maintains an above-threshold drug concentration. This is achieved by modelling the time course of drug concentration for the particular medication in question and applying a sigmoidal threshold function to the concentration values.

By including an estimate of the absorbed drug concentration, the model described here bears a certain similarity to those employed in the field of Pharmaco-Kinetics (PK) to describe the absorption and elimination rates of pharmaceuticals. Indeed, previous studies have exploited the interface between PK models and adherence to improve estimates of medication effectiveness [36, 37]. However, while PK models are concerned with the precise description of biochemical pathways and drug metabolism, our model is rather concerned with estimating the therapeutic effectiveness of treatment. Thus the parameters of our metric are specifically fitted against relevant measures of clinical outcomes, and quantify aspects of the patient’s responsiveness to treatment, rather than specific biological properties.

### Deriving a model-based measure of adherence

**Fig 2:**
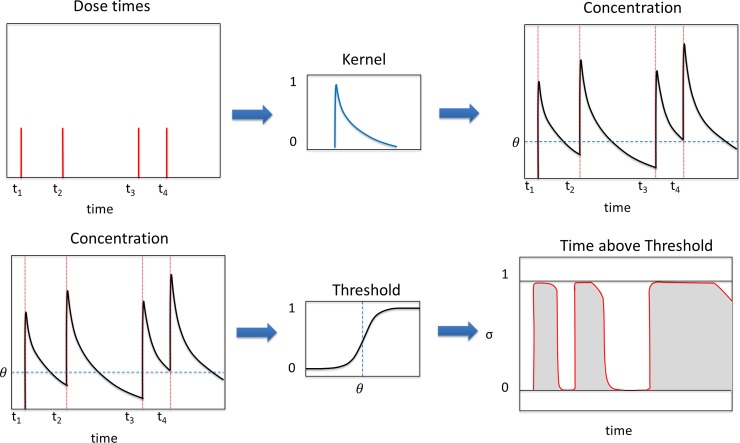
Schematic of time-above-threshold calculation. This method allows us to calculate a single robust adherence score based on timing and technique data from the monitoring device. First, the set of dose times is convolved with an exponential kernel to estimate the concentration over time (top). This concentration is then passed through a sigmoidal threshold function (bottom) and the output integrated to give the proportion of time above threshold.

We define a measure of adherence based on dose timing and technique information obtained from a remote monitoring device such as the INCA. However, it should be noted that this method is not specific to this particular device or treatment, and may potentially be applied to a range of treatments whose use can be accurately timed. A schematic of this model is shown in Fig 2.

Given a set of dose timings, {*t*_*s*_}, and technique scores {*δ*_*s*_}, we model the time course of the inhaled drug concentration, *C*(*t*). In the case of inhaled medications such as those employed here, the drug is absorbed rapidly by the targeted tissues [33, 34], and so the time-course of the concentration can be approximated by an exponential function of the form

$$C(t)\propto \sum_{t_{s}<t}\delta_{s}e^{-\alpha (t-t_{s})}$$ (1)

Where *t*_*s*_ is the set of dose times obtained from the monitoring device, and the parameter *α* represents the decay rate of the drug in question, and is related to the physiological half-life by the standard equation

$$T_{1/2}=\frac{ln2}{\alpha }.$$ (2)

In the case of other medications, where the absorption of the drug cannot be approximated as instantaneous, suitable PK profiles may be used to obtain a more appropriate estimate of *C*(*t*).

The parameter *δ*_*s*_ is a technique score, representing the proportion of the full dose received. This takes the value of 1 for correctly taken doses, and values between 0 and 1 for doses where technique errors were made. In our datasets this score is obtained from the INCA^TM^ device, which assesses the efficiency of inhalation on each inhaler use [38, 39].

Since *δ* = 1 for all correctly taken doses, independent of the actual dosage, the concentration, *C(t)* and threshold value, *θ*, are expressed in units of the prescribed dosage (see S1 Mathematical Appendix).

To determine the proportion of time spent above threshold, the concentration value is then passed through a sigmoidal threshold function:

$$\sigma (t)=\frac{1}{1+e^{-\beta (C(t)-\theta )}}$$ (3)

The form of this function matches that of the standard dose-response curve, and thus models the non-linear dependence of patient outcomes on drug concentration [30, 31, 40].

**Fig 3:**
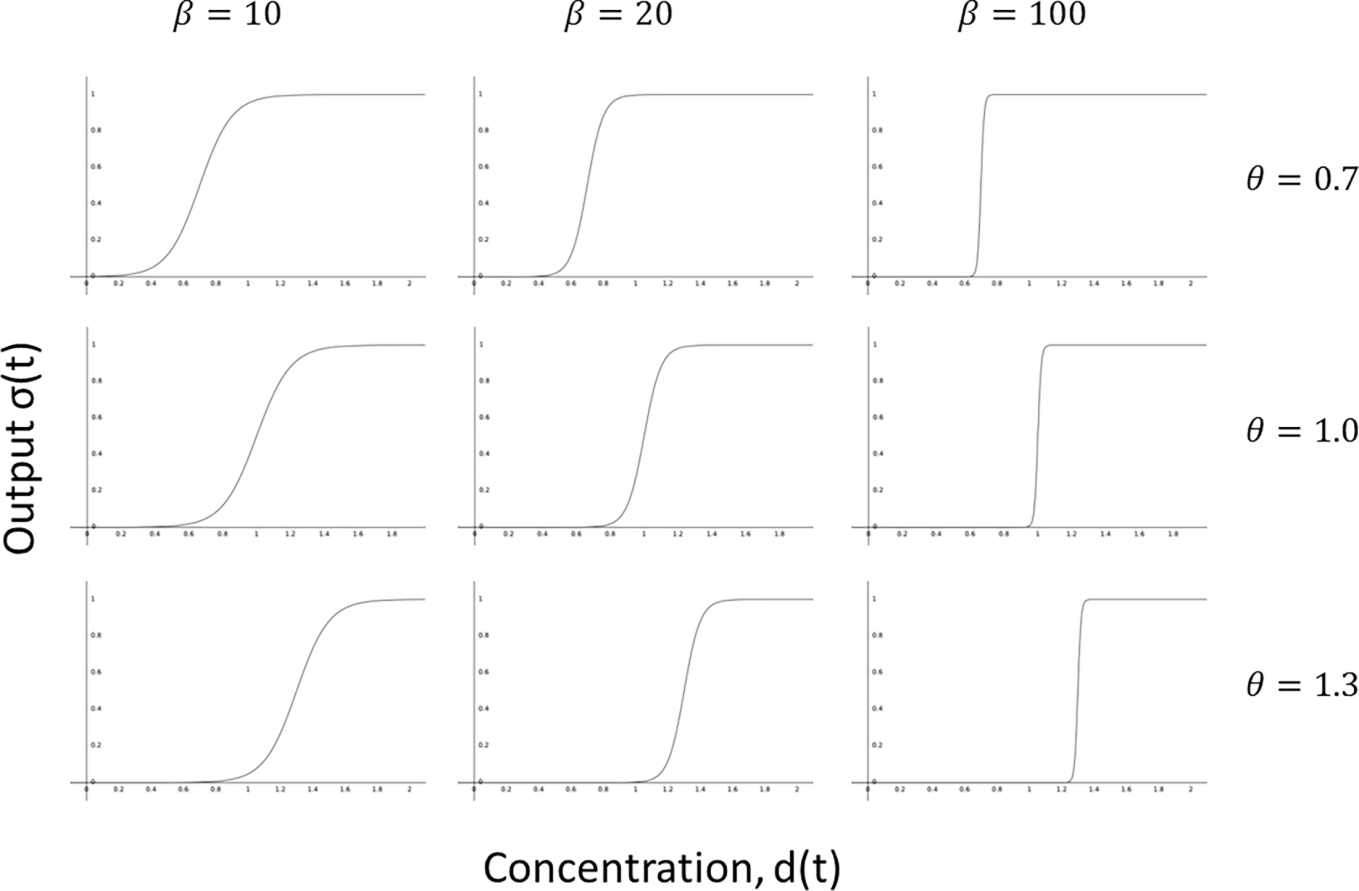
The effects of parameters *θ* and *β* on the threshold function. For large values of *β* the function becomes step-like, while for small values it is smoother, and approximately linear. Changes in *θ* shift the function to the right or left.

The parameters *θ* and *β*, of the threshold function represent the threshold value and the threshold sharpness respectively. That is, *θ* represents the target drug concentration needed to control the condition, while the parameter *β* captures how strongly the adherence score depends on the threshold. Thus, for large values of *β*, the threshold function approximates a step function, with a value of 1 when the concentration is above threshold, and 0 otherwise. For small values of *β* the transition becomes smoother, and the threshold effect is weaker (see Fig 3).

Finally, a summary adherence score for the clinically relevant time period can now be obtained by averaging the output of the threshold function over the time interval in question, giving a value *A* between 0 and 1 representing the proportion of time above threshold:

$$A(\alpha ,\beta ,\theta )=\frac{1}{T}\;\int_{0}^{T}\sigma (t)dt.$$ (4)

### Threshold concentration and required dosage

We have defined adherence in terms of the threshold concentration, *θ*, and an increment *δ*, where both *θ* and *δ* are expressed in units of the prescribed dosage. Using equations (1)-(4) we can thus derive the relationship between the threshold level and the dosage required to maintain that concentration under perfect adherence (See S1 Mathematical Appendix):

$$\delta_{r}=-\frac{\theta (e^{-h\ln2}-1)}{e^{-h\ln2}}=-\frac{\left(0.5^{h}-1\right)}{0.5^{h}}\theta$$ (5)

where *δ*_*r*_ is the required dose, expressed in units of the current dosage, and *h* is a constant corresponding to the expected number of half-lives between doses. In the case where the interval between doses equals the half-life, we get *δ*_*r*_ = *θ*, and so the threshold level can be interpreted as the proportion of the current dosage needed to maintain health. (e.g. *θ* = 0.5 would imply that half the current dosage would suffice under perfect adherence). Furthermore, given an estimate of the average adherence rate for a particular patient, this required dosage can be easily adjusted to determine a patient-specific dosage.

### Model fitting

The adherence measure described above depends explicitly on the values of the parameters *α* (drug decay rate), *δ* (technique score), *β* (threshold steepness parameter), *θ* (optimal drug threshold). While the technique parameter *δ* is a property of the specific delivery method used, the decay constant *α* and threshold parameters *β* and *θ* reflect the physiology of the cohort in question and of the condition being treated, and so are not known *a priori*. As a result, to obtain the most useful clinical measure of adherence, we can optimise our model by fitting these parameters so that the resulting adherence score gives the best prediction of patient outcomes. The optimisation is performed using standard maximum likelihood methods (see S1 Mathematical Appendix for details).

In this paper we apply our model to a population of patients with COPD. This population is of particular clinical interest for adherence studies, being characterised by poor adherence and frequent re-admission to hospital. However, due to the very high level of technique errors in this cohort (errors detected in approximately 60% of all inhaler uses), it proved difficult to robustly fit our model using this dataset. In order to avoid this problem, and obtain a sufficiently robust model which is not prone to over-fitting, we instead optimise the model using ‘training data’ from a distinct cohort of acute asthma sufferers, before validating the model against our COPD data. This has the advantage of providing a larger set of usable training data, as well as eliminating any problem due to overfitting, since the training and validation datasets are completely distinct.

It should be noted that the treatment in question in this study (ICS-LABA inhalers) contain two distinct drug components, namely a steroid component and a long acting beta-agonist, which target distinct aspects of lung disease. In our case, while LABA may have a short term effect on PEFR, we might expect the steroid component of the drug to be more predictive of longer-term measures like exacerbation rate. As a result, by choosing an outcome against which to optimise the model, we implicitly optimise the model to capture the effect of one or the other of these components.

### Datasets

#### Training data: Uncontrolled asthma cohort

The model was optimised using pre-existing adherence data and measures of clinical outcomes obtained from a clinical trial of the INCA^TM^ device in an asthma cohort [41, 42] (Table 1.). This was a randomised controlled trial for an adherence intervention using the INCA device fitted to Seretide^TM^ salmeterol/fluticasone inhalers. The training set consisted of up to three months of adherence and clinical data from 218 otherwise healthy patients with moderate to severe uncontrolled asthma. All patients were prescribed a twice-daily dose, intended to be taken at intervals of 12 hours.

**Table 1:** Summary statistics for asthma cohort.

CHARACTERISTICS	(n = 218)
Mean Age	49.2(16.5)
Sex (% Female)	64%
BMI	29.9 (7.0)
Currently smoking (%)	8%
Salmeterol/fluticasone Dose (%):	
500mcg	65%
250mcg	35%
Inhaler Proficiency Score (range 0–10)	7.5 (2,7)
DISEASE SEVERITY	
FEV_1_ (L)	2.2 (0.9)
AQLQ	3.7 (1.2)
ACT	12.1 (4.5)
PEF (L/MIN)	376.1 (135.5)
PEF (% EXPECTED)	81.6 (23.5)

The data contain two principal clinical outcomes:

- Twice daily measurements of Peak Expiratory Flow Rate (PEFR)
- Monthly recording of occurrence of Asthma Exacerbations

PEFR was measured recorded twice daily using a hand-held electronic peak flow meter. In total the dataset contained 16,934 PEFR measurements, while there were 85 self-reported exacerbations and 64 physician-defined exacerbations during the study period. Self-reported exacerbations were defined as a significant worsening of symptoms which resulted in the prescription of oral steroids. Physician-defined exacerbations were defined from analysis of PEFR recordings, and relate to a sustained clinically-significant drop in PEFR (20%). For the purposes of model fitting self-reported exacerbations were used, however analysis of exacerbation timing showed good agreement between self-report and physician defined exacerbations for this cohort.

Given these two outcome measures, we can separately optimise the model against each outcome, to obtain individual estimates of the parameters (*α*,*δ*,*β*,*θ*).

When fitting against the occurrence of adverse events, which is a binary measure covering a one-month period, the predictor used is the overall monthly adherence score (*α*,*δ*,*β*,*θ*). When fitting against daily peak flow data, we employ a moving average of daily adherence scores, with lag time *τ*, determined from the reverse correlation of peak flow against adherence.

Parameter values obtained from each of these optimisation methods are broadly similar, and so for validation purposes we consider only those estimates obtained using the monthly exacerbation data.

#### Validation data: The INCA-COPD cohort

We investigated the predictive validity of our fitted model by applying it to adherence data from a separate observational cohort of patients with COPD (INCA-COPD [43]). This study gathered 3 months of adherence data from 265 patients, of whom 204 had usable adherence data. These patients generally exhibit very poor adherence, which may be in part due to high levels of cognitive impairment and co-morbidity (Table 2). In total there were 226 self-reported exacerbations among this cohort during the three months of the study.

**Table 2:** Summary statistics for INCA-COPD study.

CHARACTERISTICS	(n = 265)
Mean Age	70.6 (9.8)
Sex (% Female)	53%
BMI	27.5 (6.6)
Median Pack Years smoked (IQR)	47.0 (47.1)
Currently smoking (%)	22%
Salmeterol/fluticasone Dose (%):	
500mcg	75%
250mcg	25%
Inhaler Proficiency Score (range 0–10)	7.6 (1.6)
DISEASE SEVERITY	
FEV_1_ (L)	1.3 (0.6)
FEV_1_ (%)	51.7 (21.3)
Cough PEF	159.6 (99.2)
CAT score	20.5 (7.9)
Number of COPD admissions in previous year	1.3 (1.7)
Median MRC dyspnoea score (IQR)	3.6 (1.1)
PERSONAL FACTORS	
Charlson Co-Morbidity	5.9 (1.8)
MoCA score (range 0–30)	20.2 (6.2)

As in the asthma study, all patients were prescribed a twice daily dose from a fluticasone/salmeterol inhaler, to be taken at regular 12 hour intervals, and had their adherence to this regimen monitored using the INCA device for a period of three months. No daily PEFR measurements were available for this cohort, and so self-reported exacerbations were used as the outcome of interest. Specifically, we use the adherence score calculated over a single month as a predictor of the number of exacerbations reported within that month, for each of the three months in the study.

## Results

### Fitted parameter values and dosages

Tables 3 and 4 below show the values of the technique parameter, *δ*, and model parameters (*α*,*β*,*θ*,*τ*) respectively. The values of *δ* shown were obtained from calibration tests of the INCA device [44].

**Table 3:** Values of parameter *δ* corresponding to particular technique errors. Results obtained from calibration tests of the INCA device [44].

Error Type	*δ*
**None**	1.0
**Low inspiratory flow rate (PIFR < 35 L/min)**	0.7
**Exhalation error (patient exhales into inhaler)**	0.5
**Low PIFR + Exhalation error**	0.35
**No blister detected (drug not released)**	0
**No inhalation detected**	0

**Table 4:** Fitted parameter values obtained using Peak Expiratory Flow Rate (PEFR) and Adverse event rate (AE rate) respectively as target outcomes.

Parameter	Peak Flow	Adverse event rate
***α***	0.062 ($T_{\frac{1}{2}}=11.2\;hrs$)	0.056 ($T_{\frac{1}{2}}=12.4\;hrs$)
***β***	63	73
***θ***	0.56	0.69
***τ***	18.2 hrs	-

Table 4 shows values of fitted parameters obtained using maximum likelihood model fitting against both target variables. In both cases the predictive power of the model showed a weak dependency on *β* within a broad range of values, (approximately 50–100) indicating that the exact slope of the threshold function is not of great importance. However in both cases, the model accuracy depended strongly on the threshold value, with optimal values of approximately 0.56 (95% C.I. 0.49–0.7) and 0.69 (0.52–0.75) for peak flow and A.E rate respectively.

Optimal values of the decay rate, *α*, corresponded in both cases to half lives in the region of 12 hours. This matches the expected interval between doses (twice daily), and so from Eq (5) we can interpret the corresponding threshold value, as the proportion of the current dosage needed under perfect adherence (for more detail on the relationship between prescribed dosage and threshold value, see S1 Mathematical Appendix).

This suggests that the currently prescribed dose is larger than needed for this particular cohort (optimal dose = 0.56 or 0.69 times the current dose), assuming good adherence.

It is interesting to note that analysis of predictors of adherence found no dependence on sex, age or other demographic factors (data not shown). This suggests that effective adherence is driven by individual behavioural or clinical factors.

### Predictive validity of new adherence model

Participants were classified according to whether or not they reported an exacerbation within a one-month period, and adherence scores from the same period were used as a predictor in a logistic regression model. As shown in Table 5, adherence scores calculated using our threshold method were found to be highly predictive of self-reported exacerbations, with a 48% reduction in odds of an exacerbation per standard deviation increase in adherence score (Odds Ratio = 0.52, p = 0.002). In particular, our adherence measure gave greatly improved prediction of exacerbations compared to the best previously known method, the AUC metric (Odds ratio 0.69, p = 0.04).

**Table 5:** Logistic regression of self-reported exacerbation against our new adherence metric (left) and the AUC metric (right) [29].

	New Metric			AUC Metric [43]		
Variable	Odds Ratio	95% C.I.	p	Odds Ratio	95% C.I.	p
Standardized Adherence	0.52	[0.34, 0.79]	0.002	0.69	[0.48, 0.99]	**0.04**
Age	0.99	[0.95, 1.02]	0.39	0.99	[0.95, 1.02]	**0.45**
Sex	1.65	[0.82, 3.31]	0.16	1.71	[0.86, 3.37]	**0.13**
Intercept	0.48	[0.03, 7.33]	0.60	0.36	[0.03, 5.25]	**0.46**

## Discussion

Recent advances in remote monitoring technology provide rich sources of data on adherence to medication [38, 45–47]. At the same time, the increased focus on data-driven medicine and the development of numerous advanced biomarkers has raised the possibility of individualised or patient-specific treatment of many chronic conditions [48–50]. However, to fully take advantage of these possibilities we require the corresponding analytical tools, which will allow us to properly exploit the complexity of these data, and provide the necessary links between treatment, adherence and outcomes.

The method described in this paper is hoped to be a significant step in this direction. Rather than considering dosing, treatment, and adherence assessment as independent processes, we hope to demonstrate that they are in fact part of a single complex relationship between patient’s behaviour and their health, and are as a result inextricably linked.

This method represents both a technical advance over previously used methods of adherence assessment, and an opportunity for the development of individualised treatments for chronic conditions such as COPD.

### Advantages over established methods

By incorporating specific information about the delivery method, effectiveness and pharmaco-kinetics of any given drug, the metric described here offers several significant advantages over previously used methods. First, it explicitly captures the exponential decay of drug concentration, while by contrast other methods of adherence employ linear summation of doses taken, or similar. Thus, this metric is sensitive to dose-timing effects which are ignored by previous methods. Second, steroid medications, such as those used in inhalers, are known to exhibit sigmoidal dose-response curves [31, 51], with the result that they are most effective only when a minimum concentration has been reached, but have little additional benefit at higher concentrations [30, 31]. These effects are specifically accounted for by the use of a sigmoidal threshold function. As a result, the time-above-threshold metric is significantly predictive of patient outcomes (see Table 5).

### Model parameters and dosage calculation

Unlike standard measures of adherence, this method is based on a parametric modelling of the patient’s response to a specific medication. Two of these parameters, *α* and *δ*, depend primarily on the specific drug and delivery method, and may be taken from the literature or established through direct measurement. However the values of the threshold parameters, *β* and *θ*, are not established and cannot be easily determined experimentally. Instead, these parameters are fitted against patient outcome data (e.g. peak-flow, number of exacerbations) and the chosen values of these parameters are defined as those for which the adherence measure is most strongly predictive of patient outcomes. In simple terms, we define our model as: “The best predictor of the outcome variable (either variations in peak flow or occurrence of exacerbations) is the amount of time the patient spends above threshold value *θ*.” We then find the value of *θ* which best fits this model.

As a result, what we obtain here is a purely *functional* measure of adherence, which is specific to a particular drug, disease, and patient cohort. In fact, rather than assess how well the patient complies with a one-size-fits-all treatment plan, it is more accurate to say that with this method we attempt to assess to what extent the patient is receiving the required level of treatment to maintain good health, given the specific condition being treated.

We are able, therefore, to derive a formula (Eq 5) which defines the optimal dosage needed to treat a condition using patient adherence and outcome data.

### Closing the loop between treatment and adherence

Recent years have seen an increase in studies of adherence, but also in a focus on behaviour-driven and data-driven approaches to medicine. Increasingly, the distinctions between these approaches are blurred. By expressing adherence in terms of parameters which are themselves determined *a posteriori* from the patients’ clinical data, we make an explicit connection between the measurement of adherence and the measurement of treatment effectiveness.

While the results presented here employ a single parameter set to describe an entire patient cohort, the relationship between the threshold parameter and the required dosage, defined by Eq 5 suggests clear possibilities for the development of more individualised treatment. Given the increasing availability of adherence monitoring data, it may soon be possible to apply this method to determine patient-specific thresholds, and hence, patient specific dosing plans.

Increasingly the use of highly sensitive biomarkers allows patients to be classified according to their individual responsiveness to treatment [48]. For example, in the case of steroid medications such as those employed here, a number of biomarkers such as Fraction of Exhaled Nitrous Oxide (FeNO), eosinophil count and urinary bromotyrosine level (BrTyr), have been identified as highly sensitive predictors of patient response [52, 53]. Given sufficiently sensitive biomarkers such as these, and precisely timed adherence data, the method described here can feasibly be employed to determine a patient specific threshold level, and thus determine on a patient-by-patient basis the dosage needed to treat conditions such as asthma and COPD. It is our intention to demonstrate this in the near future using data from a cohort of asthma patients whose adherence and response to treatment are monitored using the INCA device and repeated FeNO measurements respectively.

### Generalisation to other conditions and clinical trials

While the data used in this study are obtained from the INCA device, the method described here is a general one, which can potentially be applied to a wide range of other treatments. It may be employed with little or no alteration to other inhaled treatment regimens such as SMART [54], while more generally it can potentially be adapted for use with almost any treatment or delivery method that can be precisely monitored. For example, remote monitoring of injections in treatment of HIV and diabetes has been common for several years, while numerous methods for the remote timing of pill ingestion have been developed [45, 47, 55–57]. However, while a wealth of adherence information is available for these treatments, little or no effort has been made to apply modelling techniques such as those described here. It is our intention to develop the method described above into a more general framework combined with a software implementation, which could be applied to datasets from any of these other devices or adherence monitors.

However, perhaps the most interesting possibility for the wider application of this method is in clinical trials. It is widely acknowledged that non-adherence presents a significant problem in ambulatory drug trials, with a wide range of adherence assessments in use [58–61]. A high degree of variability in adherence among trial participants can have the effect of introducing increase variability in patient response, as well as a reduction in overall response to treatment, with a resulting decrease in statistical power [61]. As a result, larger patient numbers are required to achieve sufficient power, with corresponding increases in cost. Our adherence allows variability in adherence to be explicitly accounted for when determining the effectiveness of treatment, which has the potential to improve the sensitivity and cost-effectiveness of such trials. In addition, we describe a method to explicitly determine appropriate dosages based on the adherence data of the trial population.

### Limitations and future work

While we believe that we have demonstrated the utility of our adherence model, it must be acknowledged that the work presented here has several limitations. The most important limitation is in the paucity of outcome data for our COPD population. While we have discussed the possibility of calculating cohort-specific or patient-specific dosages, we were in fact unable to do this in the case of our population of interest, due to a lack of sufficient data on patient outcomes against which to fit the model. Similarly, while exacerbations are an important clinical outcome, patients’ reported symptoms may provide a more accurate measure of quality of life and well-being, and as such would be an equally valid outcome measure. However, due to a lack of consistent reporting of symptoms between our two datasets, it was not possible to model this outcome here.

Instead, we fitted our model using a more detailed dataset from acohort of asthma sufferers, and applied this model to predict outcomes for our COPD cohort. While less than ideal, this does has the benefit of completely avoiding the risk of over-fitting our model. Indeed, the fact that a model fitted against data from one cohort could be used to predict outcomes for an entirely different cohort tends to demonstrate the robustness of the model. Nonetheless, it would be preferable to fit the model directly against data from the target population using a sufficiently sensitive biomarker as the outcome variable–future work should address this.

This study is also limited in that we have modelled only the effect of preventative medication (ICS-LABA), and have not accounted for the use of additional reliever medications (e.g. salbutamol inhalers). It is acknowledged that many patients may compensate for poor adherence to ICS by reliance on reliever medication [62], and so a complete understanding of the relationship between adherence and outcomes would also require the monitoring of such medications. While the method described here may be easily applied to salbutamol, or other inhaled reliever medications, such data was not available to us, and so could not be included in this study. Future work should address this.

Furthermore, while we hope this paper will be of interest to those involved in the treatment of chronic disease, we appreciate that a practical implementation of this method would be of much greater utility. We are currently engaged in the production of a software package in Stata to implement this method, and have provided here Stata code for the calculation of adherence scores with an accompanying sample dataset. It is our hope that these packages will be of use in the development and testing of new treatments, as well as in clinical practice, through the provision of accurate estimates of dosages required, as well as the provision of improved feedback on adherence to patients with chronic illness.

## Supporting information

S1 Mathematical Appendix Additional information on model fitting, calculation of likelihood functions, and optimal dosage calculation. (DOCX)

S1 Dataset Minimal dataset required to reproduce primary result (Table 5). (DTA)

S2 Dataset Example raw INCA data for use with stata code (S1 Stata Code). (DTA)

S1 Stata Code Demonstration stata code for calculation of threshold adherence metric. (DO)
