# Supplementary material for: A novel statistical method for assessing effective adherence to medication and calculating optimal drug dosages
Source: PLoS One. 2018 Apr 20;13(4):e0195663. doi: 10.1371/journal.pone.0195663 (PMC5909911; doi:10.1371/journal.pone.0195663)
Supplement: S1 Mathematical Appendix — (DOCX) [file pone.0195663.s001.docx]

**Mathematical Appendix**

**Minimum Dosage Value**

We seek the minimum dose increment, δ, required to maintain concentration above threshold value θ.

We first consider the ideal case of perfect adherence, with all doses taken correctly at uniform time intervals. Let δ represent the change in concentration resulting from a correctly taken dose, which is assumed to be directly proportional to the prescribed dosage, and let τ represent the interval between doses.

Assuming a simple exponential decay, the minimum value of δ required to maintain a concentration level $C\left( t \right)\geq\theta$ is that which satisfies

$$\left( \theta+\delta\right)e^{-\alpha\tau}=\theta$$

$$\underset{\Rightarrow}{} \theta\left( e^{-\alpha\tau}-1 \right)=-\delta e^{-\alpha\tau}$$

Re-arranging for δ gives:

$$\delta=-\frac{\theta\left( e^{-\alpha\tau}-1 \right)}{e^{-\alpha\tau}}$$

Writing the decay rate, α in terms of the half-life as $\alpha=\frac{\ln2}{T_{1/2}}$ gives

$$\delta=-\frac{\theta\left( e^{-\frac{\ln2}{T_{1/2}} \tau}-1 \right)}{e^{-\frac{\ln2}{T_{1/2}} \tau}}$$

In the ideal case where doses are spaced at intervals of exactly one half-life, we have $\tau= T_{1/2}$ which gives

$$\delta=-\frac{\theta\left( e^{-\ln2}-1 \right)}{e^{-\ln2}}=\theta$$

Thus, in the ideal case we have $\delta=\theta$, where δ is proportional to the received dosage. Setting $\delta=1$ for the currently prescribed dosage, we can thus interpret the threshold parameter θ as the proportion of the current dosage required to adequately control the condition.

In the more general case where dose intervals do not equal one half-life, we can write the interval τ in terms of the half-life as $\tau=hT_{1/2}$ for some constant *h*, and again, assuming perfect adherence at regular intervals, we can write δ in terms of the threshold value as:

$$\delta=-\frac{\theta\left( e^{-h\ln2}-1 \right)}{e^{-h\ln2}}= -\frac{\left( {0.5}^{h}-1 \right)}{{0.5}^{h}}\theta$$

So for example, if we double the frequency of doses, we get $h=\frac{1}{2}$ which gives $\delta=0.41\theta$ implying that the minimum required dose is $0.41\theta$ times the current dosage.

**Maximum Likelihood Estimation**

The parameter values used in calculating adherence scores were optimised against patient outcomes by means of maximum likelihood estimation. To achieve this we must derive an expression for the likelihood function (i.e. the probability of the outcome data, written as a function of the parameters), and its derivatives with respect to each of the model parameters. This function is then maximised by means of simple gradient ascent.

In practice, since the expression for the likelihood is generally written as a product of probabilities, it is often easier to consider the logarithm of the likelihood, which can be expressed as a sum. Since in general $\log A$ increases monotonically with $A$, maximising $\log A$ also maximises $A$.

**Likelihood Maximisation for exacerbation rate:**

Given adherence score $A_{i}\left( \theta,\beta,\alpha\right)$ and number of exacerbations, $k_{i}$ for each patient $i$, we consider exacerbations as a

Poisson process, where the probability of exacerbation is given by $(1-A_{i})$.

Thus, we can write the likelihood function as

$$L\left( k | A\left( \alpha, \beta,\theta\right) \right)= \prod_{i:k_{i}>0} \left( 1-A_{i} \right)^{k_{i}}\prod_{i:k_{i}=0} A_{i}$$

Taking the log of both sides we get the log-likelihood:

$$\log L\left( k | A\left( \alpha, \beta,\theta\right) \right)= \sum_{i:k_{i}>0} k_{i}\log(1-A_{i})+\sum_{i:k_{i}=0} \log A_{i}$$

We next find the derivatives of the log-likelihood with respect to the parameters $\theta, \alpha, \beta$:

$$\frac{\partial\log L}{\partial\theta}= -\sum_{i:k_{i}>0} \frac{k_{i}}{(1-A_{i})}\frac{\partial A_{i}}{\partial\theta}+\sum_{i:k_{i}=0} \frac{1}{A_{i}}\frac{\partial A_{i}}{\partial\theta}$$

And likewise for α and β.

It remains to find the derivatives of $A_{i}$w.r.t. $\theta, \alpha, \beta$

From Equations (1-4) we have:

$$A\left( \alpha,\beta,\theta\right)=\frac{1}{T} \int_{0}^{T} \sigma\left( t \right)dt$$

Where:

$$\sigma\left( t \right)= \frac{1}{1+ e^{-\beta(C\left( t \right)- \theta)}}$$

And

$$C\left( t \right)= \sum_{t_{s}<t} \delta e^{-\alpha\left( t-t_{s} \right)}$$

Differentiating with respect to $\alpha$ gives:

$$\frac{\partial A_{i}}{\partial\alpha}= \frac{1}{T} \int_{0}^{T} \frac{\partial\sigma\left( t \right)}{\partial\alpha}dt= \frac{1}{T} \int_{0}^{T} \frac{\partial\sigma\left( t \right)}{\partial C\left( t,\alpha\right)}\frac{\partial C(t,\alpha)}{\partial\alpha}dt$$

Where:

$$\frac{\partial\sigma\left( t \right)}{\partial C\left( t,\alpha\right)}= \frac{\beta e^{-\beta(C\left( t \right)- \theta)}}{\left( 1+ e^{-\beta\left( C\left( t \right)- \theta\right)} \right)^{2}}$$

And

$$\frac{\partial C\left( t,\alpha\right)}{\partial\alpha}=- \sum_{t_{s}<t} \left( t-t_{s} \right)\delta_{s}e^{-\alpha\left( t-t_{s} \right)}$$

To give:

$$\frac{\partial A_{i}}{\partial\alpha}= -\frac{1}{T} \int_{0}^{T} \sum_{t_{s}<t} \left( t-t_{s} \right)\delta_{s}e^{-\alpha\left( t-t_{s} \right)}\frac{\beta e^{-\beta(C\left( t \right)- \theta)}}{\left( 1+ e^{-\beta\left( C\left( t \right)- \theta\right)} \right)^{2}}dt$$

Likewise for $\theta$we have:

$$\frac{\partial A_{i}}{\partial\theta}= \frac{1}{T} \int_{0}^{T} \frac{\partial\sigma\left( t \right)}{\partial\theta}dt= = - \frac{1}{T} \int_{0}^{T} \frac{\beta e^{-\beta(C\left( t \right)- \theta)}}{\left( 1+ e^{-\beta\left( C\left( t \right)- \theta\right)} \right)^{2}}dt$$

And for $\beta$:

$$\frac{\partial A_{i}}{\partial\beta}= \frac{1}{T} \int_{0}^{T} \frac{\partial\sigma\left( t \right)}{\partial\beta}dt= = - \frac{1}{T} \int_{0}^{T} \frac{(d\left( t \right)-\theta)e^{-\beta(C\left( t \right)- \theta)}}{\left( 1+ e^{-\beta\left( C\left( t \right)- \theta\right)} \right)^{2}}dt$$

Thus we can find an expression for the gradient of the log-likelihood with respect to the three parameters:

$$\nabla(\log L)=( \frac{\partial\log L}{\partial\alpha},\frac{\partial\log L}{\partial\beta}, \frac{\partial\log L}{\partial\theta} )$$

And the maximum likelihood estimate for the three parameters is found by simple gradient ascent.
